# Passive radiative cooling materials for extreme weather: mechanisms, design strategies, and application advances

**DOI:** 10.1039/d6ra04070e

**Published:** 2026-07-21

**Authors:** Jiaxing Du, Yanpei Wu, Shaohui Miao

**Affiliations:** a Meteorological Bureau of Tengchong City Tengchong Yunnnan 679100 China; b China Mobile Group Yunnan Co., Ltd Tengchong Branch Tengchong Yunnnan 679100 China; c Yunnan Institute of Meteorological Sciences, Research Center for Disastrous Weather in Hengduan Mountain and Low-latitude Plateau, Yunnan Meteorological Service & Chinese Academy of Meteorological Sciences Kunming Yunnan 650034 China miaoshaohui001@163.com

## Abstract

Passive radiative cooling has emerged as a promising zero-energy thermal management strategy for reducing cooling demand under increasingly severe climate conditions. This review critically examines radiative cooling materials for extreme-weather environments, where practical performance is constrained by atmospheric humidity, dust and soiling, ultraviolet aging, moisture ingress, and seasonal overcooling. Reported benchmark systems now reach solar reflectance of approximately 0.95–0.98, mid-infrared emittance of approximately 0.90–0.96, daytime sub-ambient temperature drops of about 4.5–4.9 °C, and net cooling powers of about 93–117 W m^−2^ under favorable clear-sky conditions. However, field data show that hot-humid climates can suppress or eliminate true sub-ambient cooling, shifting the practical benefit toward reduced heat gain rather than idealized temperature depression. We therefore organize the field around three linked questions: how optical mechanisms define the theoretical cooling limit, how scalable polymer and particle architectures trade spectral performance against manufacturability, and how durability, soiling, and adaptive operation determine outdoor service life. By connecting photonic design, polymer chemistry, field performance, and deployment barriers, this review identifies the material families and research gaps most relevant to building-scale and climate-resilient cooling.

## Abstract 2

This review explains how passive radiative cooling materials reflect sunlight and emit heat to outer space.

## Graphical abstract

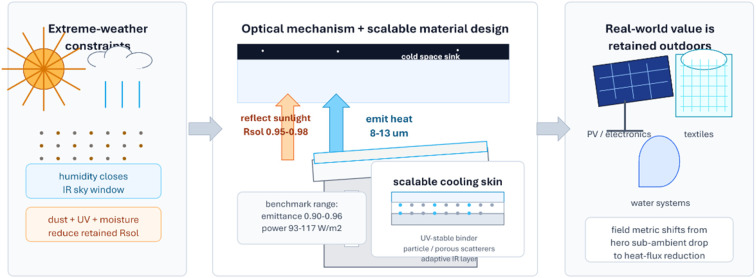

## 1. Introduction

The 21st century is defined by a dual crisis: accelerating climate change and escalating global energy demand. These two challenges are inextricably linked in a dangerous positive feedback loop, particularly concerning space cooling. The rising frequency, intensity, and duration of extreme weather events, especially heatwaves, have made access to cooling a matter of public health and economic stability.^1^ In response, the demand for conventional air conditioning (AC) is projected to triple by 2050. This reliance on vapor-compression technology is deeply problematic; it is energy-intensive, accounting for a significant portion of global electricity consumption, which in turn exacerbates greenhouse gas emissions. Furthermore, AC units function by pumping heat from a building's interior to the immediate exterior, acting as a major contributor to the urban heat island (UHI) effect, which further increases ambient temperatures and drives even greater cooling demand. This cycle underscores the urgent, global need for disruptive, zero-energy, and zero-emission cooling technologies that can function at scale.

**Fig. 1:**
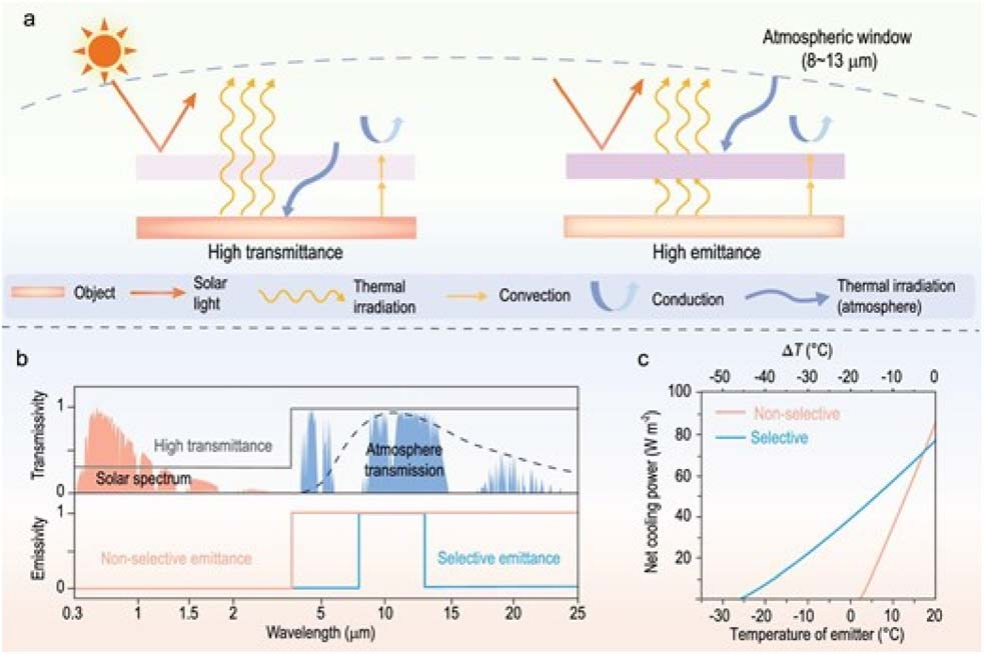
(a) Schematic heat-transfer process of the radiative cooling emitter. (b) Solar spectrum (orange-shaded area) and the atmosphere transmission spectrum in the mid-IR wavelength range (blue-shaded area). (c) Functional relationship between net cooling power, temperature of emitter and Δ*T*, reproduced from ref. 5 with permission from Oxford Academic, copyright 2023.

Passive radiative cooling (PRC) has emerged as one of the most promising technologies to meet this challenge. The fundamental mechanism of PRC is elegantly simple, leveraging the coldness of outer space (approximately 3 K) as a thermodynamic sink. A material designed for PRC must simultaneously satisfy two rigorous optical requirements.^2^ First, it must act as a near-perfect broadband mirror to incoming solar irradiance, exhibiting extremely high solar reflectance (*R*_sol_ > 0.95) across the entire solar spectrum (0.3–2.5 µm) to minimize or negate solar heat gain. Second, it must act as a highly efficient thermal emitter, possessing high selective thermal emittance (*ε*_atm_ > 0.90) precisely within the primary atmospheric transparency window (8–13 µm). This spectral window is the “cooling channel”, where the atmosphere is largely transparent to long-wave infrared (LWIR) radiation. By reflecting sunlight while simultaneously radiating its own thermal energy through this window to deep space, a PRC material can passively and continuously cool, even achieving temperatures several degrees below the ambient air temperature (*T*_amb_) under direct sunlight.^3^ To better illustrate these interrelated optical and thermal processes, Fig. 1 depicts the energy exchange pathways governing passive radiative cooling. The schematic clarifies how the emitter simultaneously reflects incident solar radiation and dissipates mid-infrared thermal energy through the atmospheric window.^4^

While foundational studies have unequivocally proven the PRC concept, their success has largely been demonstrated under idealized conditions: clear skies, moderate temperatures, and, most critically, low atmospheric humidity.^6^ The true test of PRC as a globally viable solution for climate adaptation lies in its performance under duress during the very extreme weather events when cooling is most desperately needed.^7^ This review provides a critical analysis of the field, framed by the challenge of extreme weather. This challenge is twofold. First, in hot, humid (tropical and sub-tropical) climates, high concentrations of precipitable water vapor (PWV) in the atmosphere severely absorb and re-emit infrared radiation within the 8–13 µm window, effectively “closing” the cooling channel, suppressing cooling power, and making sub-ambient temperatures nearly unattainable.^3^ Second, in hot, arid (desert) climates, while the atmospheric conditions are ideal for cooling, the materials face severe practical and engineering challenges from dust and sand soiling, which rapidly degrades solar reflectance, and extreme diurnal temperature swings that test material durability.^8^

This review moves beyond a simple survey of passive radiative cooling materials by asking which designs remain viable when the sky is humid, the surface is dirty, the polymer binder is exposed to ultraviolet radiation, and the same envelope must operate across seasons. Its novelty is to treat extreme weather as the organizing criterion rather than as an application afterthought, and to connect optical mechanisms, polymer chemistry, durability, and field performance in one critical framework. Unlike prior reviews that primarily classify emitters by material type or spectral design, this article evaluates whether each technology family can satisfy four deployment requirements simultaneously: high solar reflectance, selective atmospheric-window emission, low-cost scalable fabrication, and long-term environmental stability.

## 2. Advanced mechanisms and theoretical limits in non-ideal environments

The performance of a passive radiative cooling system is not an intrinsic property of the material alone, but rather a dynamic interplay between the material's optical properties and its surrounding environment. The theoretical maximum cooling power (*P*_net_) is defined by the net heat flux leaving the surface. This is a balance between the power radiated by the surface (*P*_rad_), the power absorbed from solar irradiance (*P*_solar_), and the power absorbed from downward atmospheric radiation (*P*_atm_). To achieve sub-ambient cooling, *P*_rad_ must exceed the sum of *P*_solar_ and *P*_atm_. While minimizing *P*_solar_ is a straightforward material challenge of maximizing solar reflectance, minimizing *P*_atm_ is a far more complex problem, as it is dictated by environmental conditions beyond the material's control.^9^ The atmosphere, even on a clear day, is not perfectly transparent; it radiates heat downward, and this radiation is the fundamental thermodynamic limit to cooling.^10^

**Fig. 2:**
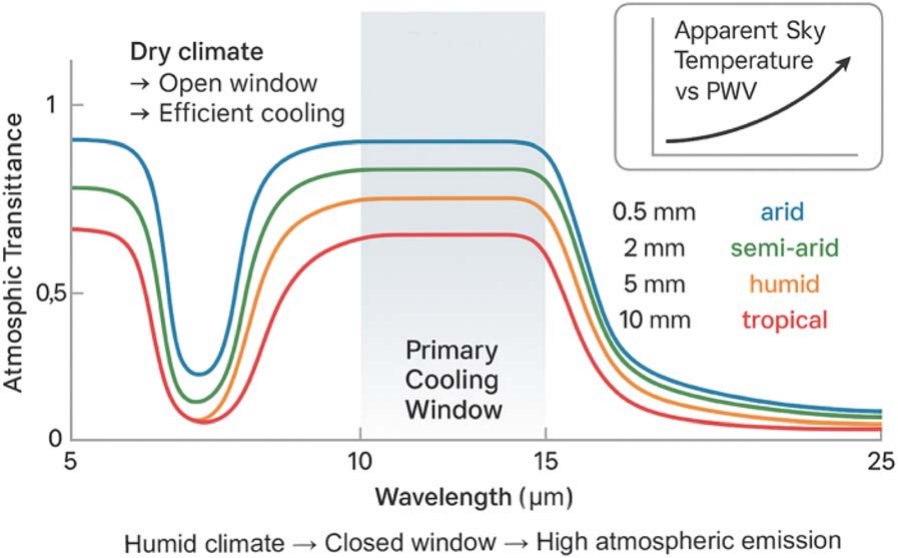
Atmospheric transmittance *vs.* precipitable water vapor (humidity). This graph illustrates the fundamental physical challenge of PRC in humid climates. The atmospheric transmittance in the primary (8–13 µm) and secondary (16–25 µm) cooling windows is plotted for different levels of precipitable water vapor (PWV). Under dry conditions (low PWV, *e.g.*, arid climates), the 8–13 µm window is wide and deep, allowing for high cooling power. As humidity increases (high PWV, *e.g.*, tropical climates), water vapor absorption bands increasingly “close” this window, drastically reducing atmospheric transparency and suppressing the net cooling potential.

The greatest real-world constraint on this limit is humidity. The primary atmospheric transparency window (8–13 µm) exists precisely because molecules like O_2_, N_2_, and CO_2_ do not have strong vibrational absorption modes in this range. Water vapor (H_2_O), however, is a powerful greenhouse gas with a broad absorption spectrum that encroaches significantly on this window. In hot and humid climates, the high concentration of precipitable water vapor (PWV) dramatically increases the atmosphere's opacity and, consequently, its downward thermal emission (*P*_atm_). This atmospheric radiation, originating from the warm, moist air mass, effectively “closes” the cooling channel. As *P*_atm_ increases, the net cooling power (*P*_net_) plummets, making sub-ambient cooling thermodynamically difficult or impossible in many tropical and subtropical regions, even with a perfect PRC material. As illustrated in Fig. 2, the influence of atmospheric humidity on the radiative cooling window is profound. The figure demonstrates how increasing precipitable water vapor (PWV) progressively narrows the 8–13 µm atmospheric transmittance window, effectively raising the sky's apparent emissive temperature. Under arid conditions (PWV < 1 mm), the window remains open, permitting efficient thermal radiation to space. In contrast, at PWV > 5 mm—typical of humid tropical regions—the window nearly closes, leading to strong reabsorption of emitted infrared photons.^11^ This quantitative relationship underscores why identical PRC materials can achieve sub-ambient cooling at night in deserts but fail under coastal humidity. It also highlights the need for hybrid strategies,^12^ such as photonic-cavity structures or hygroscopic-coupled layers, to mitigate radiative losses in moist atmospheres and preserve cooling efficacy under non-ideal environmental conditions.^13^ This humidity-induced degradation is arguably the single greatest barrier to the global, equitable deployment of PRC technologies, motivating a search for advanced mechanisms to overcome these limits.^14^

**Fig. 3:**
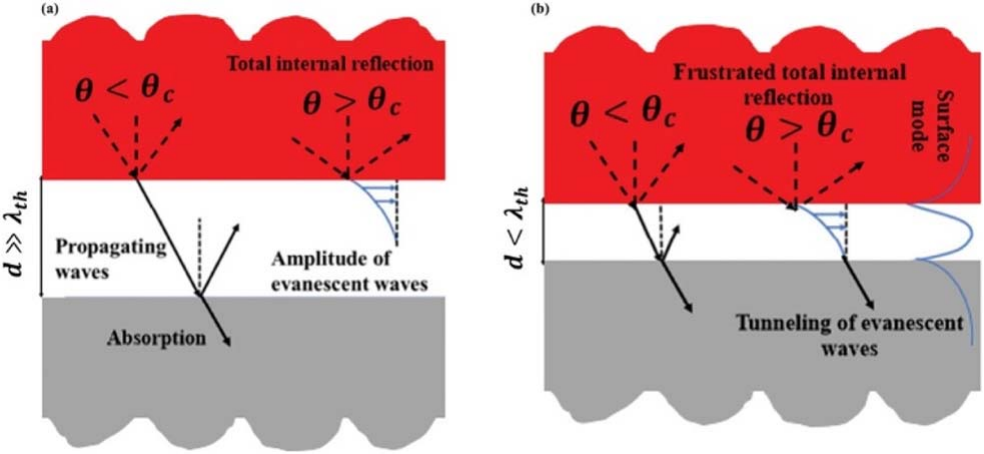
Mechanisms of thermal emission between two objects (a) far-field regime: the gap between the objects is greater than thermal wavelength. (b) Near-field regime: the gap between the objects is less than thermal wavelength, reproduced from ref. 23 with permission from Wiley & Son, copyright 2025.

One of the most theoretically powerful mechanisms for heat transfer, which bypasses environmental limitations, is near-field radiative heat transfer. In the far-field, which governs terrestrial PRC, heat transfer is limited by the Stefan–Boltzmann law for blackbody radiation and is carried by propagating electromagnetic waves. However, when two objects are brought to a separation distance (*d*) smaller than the characteristic thermal wavelength (*λ*_T_, approximately 10 µm at room temperature), a different physical regime dominates. In this near-field regime, evanescent waves—electromagnetic fields that are confined to the surfaces of the materials and decay exponentially.^15^ This tunneling of evanescent modes, particularly surface phonon-polaritons, opens a new, high-flux channel for heat transfer that can exceed the far-field blackbody limit by several orders of magnitude.^16^ While theoretically potent, the practical applications of near-field cooling are limited to nanoscale thermal management, such as cooling microelectronics or nanophotonic devices, where the required sub-micron gap separations are physically achievable.^17^ It is not a viable mechanism for cooling large-scale objects like buildings or people, which remains a fundamentally far-field challenge.^18^ To better visualize these distinct regimes of radiative heat transfer, Fig. 3 schematically illustrates the transition from the far-field to the near-field. In panel (a), heat exchange occurs *via* propagating electromagnetic waves across a macroscopic gap, constrained by the Stefan–Boltzmann limit.^19^ In contrast, panel (b) depicts the near-field regime, where the sub-wavelength separation enables evanescent waves to tunnel between surfaces, dramatically enhancing radiative flux.^20^ This figure underscores how confinement effects and surface phonon-polariton coupling can amplify energy transfer by several orders of magnitude.^21^ Such enhancement, while theoretically appealing, highlights a trade-off between performance and scalability—an essential consideration in designing next-generation thermal management systems that seek to combine near-field efficiency with macroscopic applicability.^22^

**Fig. 4:**
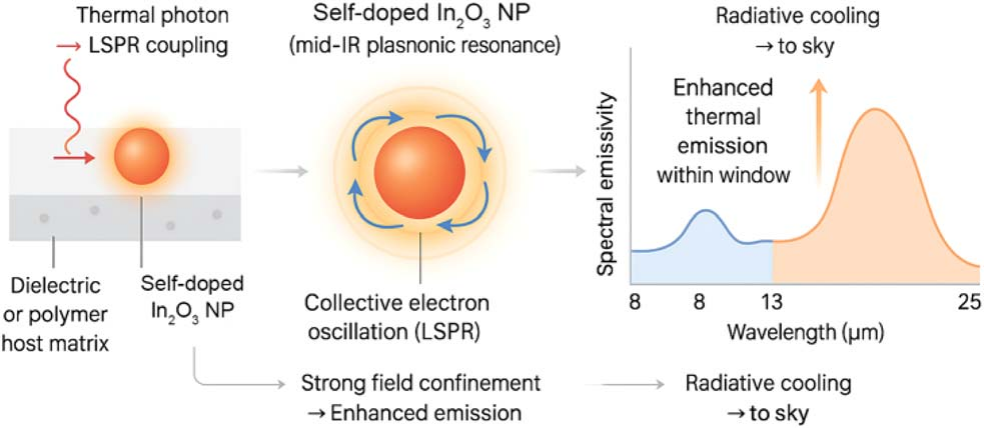
Schematic of plasmon-enhanced infrared emission. This diagram illustrates the mechanism for enhancing selective thermal emission. A semiconductor nanoparticle (*e.g.*, self-doped In_2_O_3_ or TiN) is embedded in a polymer matrix. The nanoparticle is engineered to host a localized surface plasmon resonance (LSPR) at mid-infrared frequencies. This resonance strongly couples with thermal energy, leading to a dramatic and selective enhancement of thermal emittance within the atmospheric window (8–13 µm), thereby boosting the net cooling power.

Given that terrestrial applications are confined to the far-field, research has focused on enhancing emission efficiency within the imperfect atmospheric window by engineering material resonances. Phononic resonances are a primary tool. The high infrared emissivity of many dielectrics, such as silica (SiO_2_), is due to strong molecular vibrations and the resulting phonon-polariton resonances that align with the 8–13 µm window. A more advanced approach utilizes plasmonic resonances, which arise from the collective oscillation of electrons. While metals are broadband reflectors in the IR, doped semiconductor nanoparticles, such as self-doped indium oxide (In_2_O_3_), can be engineered to exhibit strong localized surface plasmon resonance (LSPR) in the mid-infrared. As demonstrated in recent work, by controlling the nanoparticle's doping level, this LSPR can be precisely tuned to create a broad, powerful absorption/emission peak. This strategy is exceptionally effective because the plasmon-induced peak is broad enough to cover both the primary (8–13 µm) and secondary (16–25 µm) atmospheric windows, a feat that is difficult to achieve with phononic materials alone. This LSPR-enhancement pushes the material's emissive properties closer to the theoretical maximum, maximizing heat dumping through all available atmospheric “cracks”. This concept is visually summarized in Fig. 4, which schematically depicts the principle of plasmon-enhanced infrared emission. The figure shows how doped semiconductor nanoparticles embedded in a dielectric or polymer host can generate a localized surface plasmon resonance (LSPR) that overlaps with the mid-infrared atmospheric window. Through this coupling, thermal photons are funneled into resonant plasmonic modes, significantly amplifying the spectral emissivity. Importantly, the spatial confinement of these oscillations enables strong field enhancement and broadband tunability, which together bridge the gap between narrow phonon resonances and the broader spectral demands of real-world cooling. This visual framework clarifies how careful plasmonic engineering transforms otherwise reflective or weakly emissive materials into highly efficient radiative coolers capable of approaching near-ideal thermal performance.

**Fig. 5:**
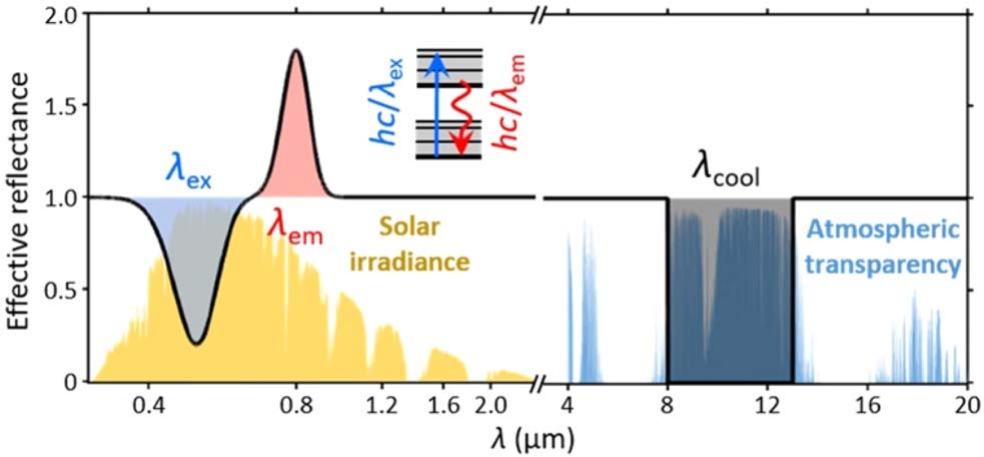
Spectral operation of a photoluminescent colored radiative cooler. The blue-shaded band marks the high-energy solar photons absorbed by the photoluminescent component; the red-shaded band marks the Stokes-shifted photons re-emitted at longer wavelength; the AM1.5 curve represents normalized incident solar irradiance; and the atmospheric-transmittance background indicates the infrared escape window. The useful cooling contribution comes from diverting absorbed visible/near-UV energy into radiative emission rather than non-radiative thermalization, reproduced from ref. 26 with permission from ACS Publications, copyright 2022.

Photoluminescent radiative cooling introduces an additional heat-management pathway beyond solar reflection and mid-infrared thermal emission. In a conventional colored surface, absorbed blue or ultraviolet photons relax non-radiatively and become phonons, increasing the material temperature. In a photoluminescent material, absorbed photons create excited electronic states that can emit lower-energy photons through Stokes-shifted photoluminescence rather than dissipating all energy as heat.^24,25^Fig. 5 should therefore be read as an energy–flow diagram as well as a spectrum: the blue-shaded region denotes the absorbed high-energy solar photons; the red-shaded region denotes re-emitted photoluminescent photons; the AM1.5 curve indicates incoming solar irradiance; and the atmospheric-transmittance background indicates whether emitted thermal radiation can escape through the sky window. The key design challenge is not simply to make a colored coating bright, but to maximize photoluminescent quantum yield, minimize non-radiative relaxation, and avoid spectral overlap that would reabsorb emitted photons inside the coating. This mechanism is attractive for architectural and textile use because it addresses the color limitation of conventional white coolers, but its present limitation is durability: quantum dots, dyes, and polymer hosts must retain optical efficiency under ultraviolet exposure, oxygen, moisture, and outdoor thermal cycling.

## 3. Material design strategies and the scalability debate

The development of passive radiative cooling materials has been characterized by a fundamental, persistent tension between two competing philosophies. The first is a “performance-first” approach, driven by physics and photonics, which seeks to achieve near-perfect spectral control to push cooling power to its theoretical limits.^27^ The second is a “scalability-first” approach, driven by chemical engineering and manufacturing, which prioritizes low cost, ease of fabrication, and compatibility with existing industrial processes, accepting a trade-off in ultimate cooling performance.^28^ This scholarly and industrial debate has created three distinct, and at times conflicting, families of PRC materials: (1) photonic metamaterials, (2) nanoparticle-based composites and paints, and (3) biomimetic and biopolymer structures. A fourth, emerging category of “scalable metamaterials” now seeks to resolve this conflict.

**Fig. 6:**
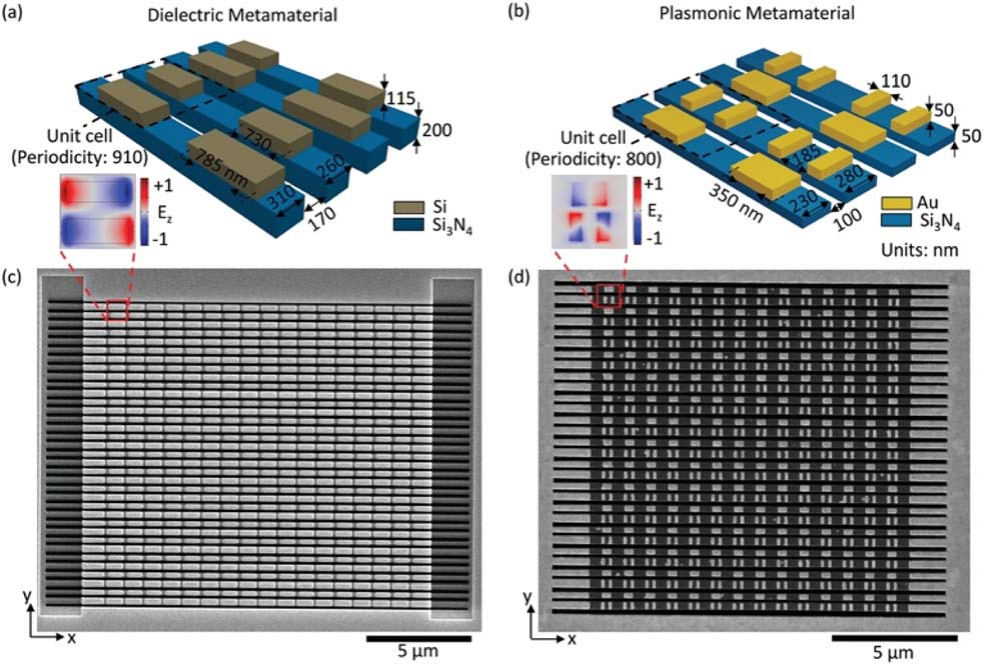
Nanomechanical photonic metamaterials. Structural schematic and SEM images of planar (a and c) dielectric and (b and d) plasmonic metamaterials fabricated on silicon nitride membranes. Insets show the resonant field distributions excited by *x*-polarized incident light at wavelengths of (a) 1550 and (b) 1310 nm, reproduced from ref. 39 with permission from ACS Publications, copyright 2022.

The “performance-first” paradigm is best exemplified by photonic metamaterials and multilayer dielectric stacks. Pioneered by research groups such as those of Fan^29^ and Raman, these materials are not random mixtures but meticulously engineered structures. They consist of multiple, nanometer-thin layers of different materials (*e.g.*, HfO_2_, SiO_2_, Ag, Ge) deposited in a precise sequence.^30^ By controlling the thickness and refractive index of each layer, it is possible to create complex interference effects that sculpt the material's optical properties, creating a photonic bandgap that results in near-perfect reflection (*R*_sol_ > 97%) of sunlight while simultaneously enhancing thermal emission in the atmospheric window.^29^ Other designs use sub-wavelength resonators, such as metamaterial pillar arrays, to achieve similar spectral precision. Fig. 6 illustrates representative examples of nanomechanical photonic metamaterials that embody the “performance-first” philosophy. As shown, planar dielectric and plasmonic architectures fabricated on silicon nitride membranes feature sub-wavelength resonators that confine and manipulate light with extreme precision.^31^ The resonant field distributions at 1550 nm and 1310 nm reveal strong localized electromagnetic enhancement within the patterned units, confirming the engineered coupling between geometry and optical response.^32^ These structures demonstrate how nanoscale control over thickness, periodicity, and refractive index can tailor both reflection and emission spectra far beyond what disordered composites can achieve.^33^ However, while such configurations achieve near-ideal spectral selectivity, their reliance on vacuum deposition and nanoscale lithography underscores the scalability barrier that continues to separate laboratory prototypes from deployable thermal-management coatings.^34^ This contrast visually reinforces the trade-off central to the ongoing debate between performance and manufacturability.^35^ The primary argument for these structures is their unparalleled performance; they have consistently demonstrated the highest cooling powers and deepest sub-ambient temperature drops, serving as benchmarks for the entire field.^36^ The core argument against them, however, is their profound lack of scalability.^37^ Fabrication relies on capital-intensive, low-throughput techniques like physical vapor deposition (PVD), plasma sputtering, or e-beam lithography which processes that are ideal for silicon chips but economically non-viable for coating the thousands of square meters of a building roof.^38^

**Fig. 7:**
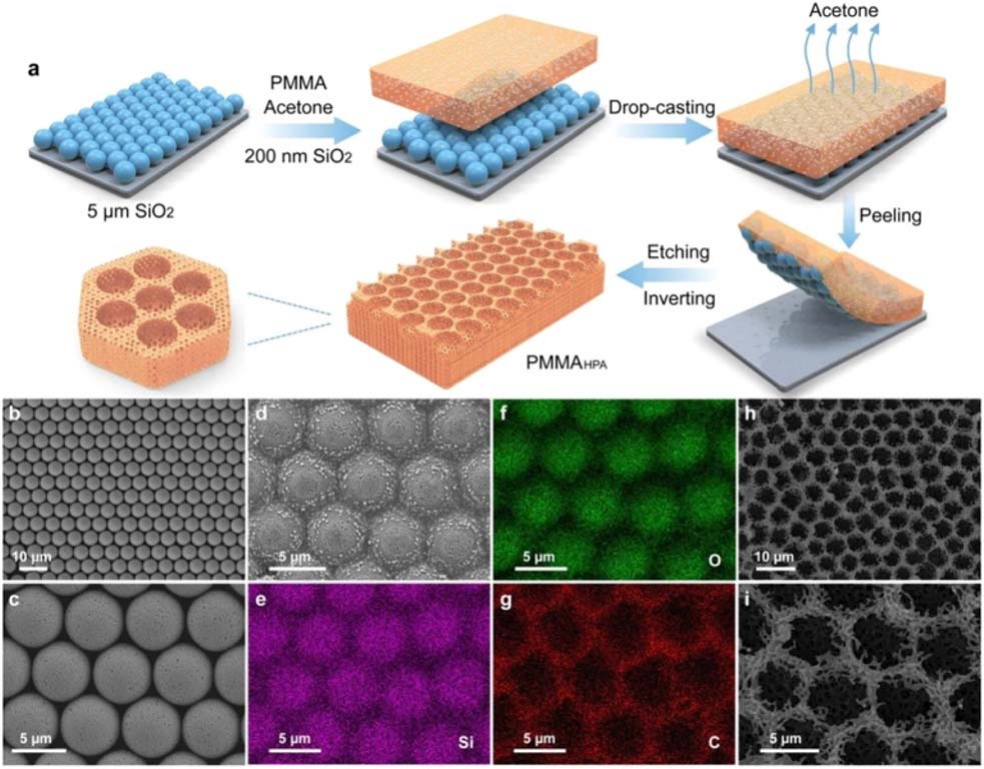
(a) Schematic illustration of the fabrication of PMMA_HPA_ with a hierarchically porous array. (b and c) SEM micrographs of hexagonally close-packed monolayer SiO_2_ templates. (d) SEM micrograph of PMMA/SiO_2_ composite. (e–g) EDS elemental mappings of Si, O, and C in (d), showing the randomly distributed SiO_2_ nanospheres and regularly distributed SiO_2_ microspheres of the composite. (h and i) SEM micrographs of PMMA_HPA_ showing an ordered symmetrical micropores array made of hierarchical randomized nanopores, reproduced from ref. 47 with permission from Springer Nature, copyright 2021.

In direct opposition stands the “scalability-first” approach, embodied by nanoparticle-based composites and cool paints. These materials typically combine a polymer binder, which is chosen for solar transparency, adhesion, processability, and intrinsic infrared emission, with dielectric particles such as TiO_2_, BaSO_4_, SiO_2_, or CaCO_3_.^40,41,61^ Their high solar reflectance is not produced by coherent interference as in multilayer stacks, but by multiple Mie scattering events from particles distributed through the binder.^42,43^ Mie scattering depends on the size parameter *x* = 2π*r*/*λ* and on the refractive-index contrast between the particle and the polymer binder; particles with diameters comparable to visible and near-infrared wavelengths scatter sunlight strongly, while an optimized size distribution broadens scattering across the solar spectrum. The binder is therefore chemically important: it must maintain a low extinction coefficient in the solar band, provide vibrational modes that contribute to 8–13 µm emission, and tolerate high particle loading without losing adhesion, flexibility, or weather resistance. Fig. 7 visualizes this principle in a hierarchically porous PMMA-SiO_2_ composite, where micro- and nanoscale features increase solar backscattering while the polymer/silica chemistry retains mid-infrared emissivity.^44–47^ At the binder level, the polymer is not merely a passive host: Si–O–Si, C–F, C=O, C–O–C and C–H vibrations determine whether the matrix remains transparent in the solar band while emitting strongly in the 8–13 µm atmospheric window. Fluorinated and siloxane-rich binders generally offer strong weatherability and low surface energy, whereas acrylic, carbonate, or ester-containing binders can provide useful processability but require careful stabilization against UV-induced oxidation. This chemistry-performance coupling explains why the best scalable coatings are not simply high-particle-load paints; they are composite optical systems in which particle size, binder refractive index, vibrational absorption, surface energy, and mechanical integrity must be co-optimized.

**Fig. 8:**
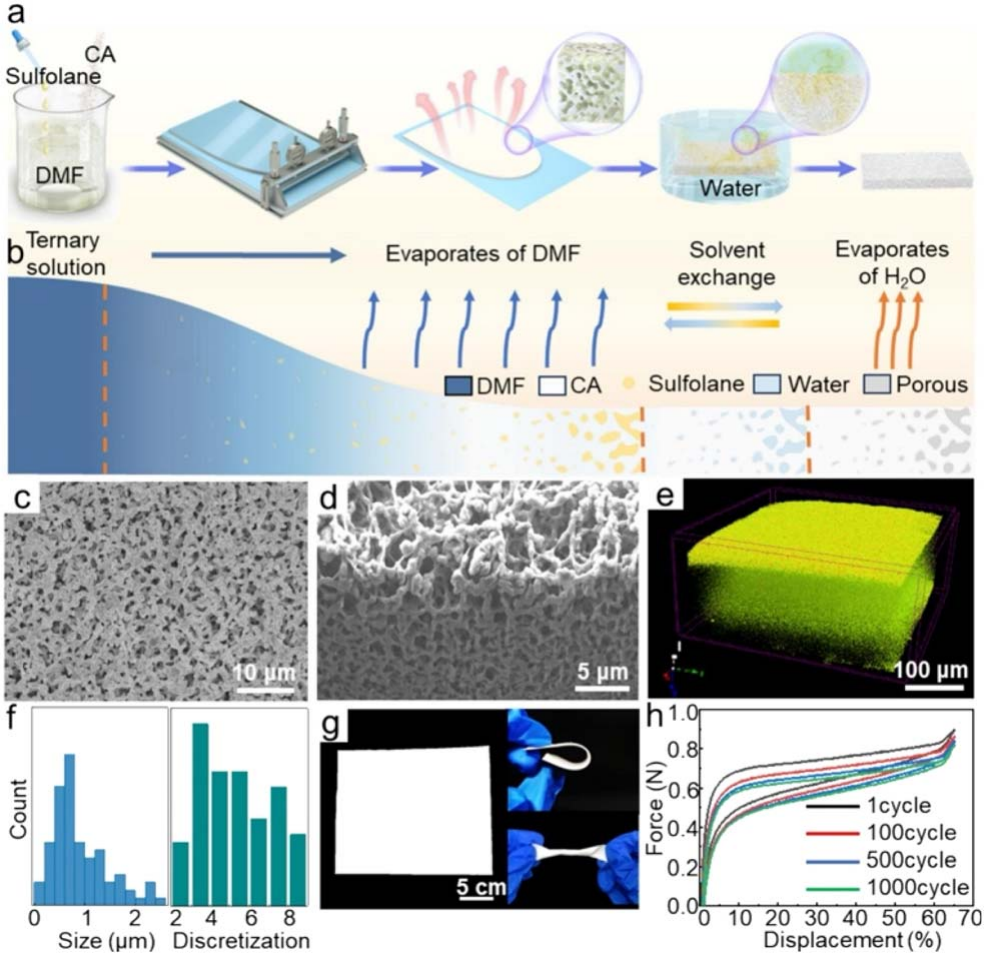
Hierarchically porous aerogel polymer metamaterial (HAPM) fabricated by surface-tension-assisted evaporation-induced phase separation (ST-EIPS). (a) Process schematic for forming the HAPM. (b) Formation mechanism showing solvent evaporation, phase separation, and pore development. (c) SEM image of the upper surface. (d) FIB-SEM cross-section showing through-thickness porosity. (e) Nano-CT three-dimensional reconstruction of the pore network. (f) Pore-size distribution and dispersion, which define the scattering-center population. (g) Photographs showing appearance and flexibility. (h) Bending curves after 1, 100, 500, and 1000 cycles, indicating mechanical retention under repeated deformation, reproduced from ref. 42 with permission from Elsevier, copyright 2025.

For a time, the field seemed defined by an intractable choice: high photonic performance or low manufacturing cost. Scalable metamaterials provide a partial resolution by using disordered rather than lithographically periodic structures. Randomly distributed dielectric spheres or hierarchical pores create a photonic-glass-like network in which many scattering paths suppress solar absorption, while the polymer and inorganic phases supply mid-infrared vibrational emission.^48–50^ The HAPM example in Fig. 8 illustrates this convergence. ST-EIPS (surface-tension-assisted evaporation-induced phase separation) generates connected micro- and nanopores rather than a single uniform pore size; SEM, FIB-SEM, and nano-CT panels show the surface, cross-section, and three-dimensional pore network; the pore-size-distribution panel quantifies the dispersion of scattering centers; and the bending-cycle curves test whether the porous architecture survives repeated mechanical deformation.^42,51,52^ The critical point is that such materials are scalable only if the optical disorder is reproducible and if the porous structure resists collapse, contamination, and moisture uptake during outdoor service.

A final, rapidly emerging design strategy is driven by sustainability: the use of biomimetic and biopolymer structures. Materials like cellulose (nanofibers, wood)^53^ and silk are drawing intense interest because they are abundant, biodegradable, and non-toxic. Their cooling properties are not an engineering afterthought but an intrinsic feature.^54^ The hierarchical nanostructure of cellulose nanofibers, for example, is highly effective at scattering sunlight,^55^ while the C–O and C–H molecular bonds within the cellulose polymer itself are powerful, broadband IR emitters. Researchers have leveraged this by engineering “cooling wood”, where wood is delignified and densified, turning its natural cellulose structure into a high-performance PRC material.^56^ Similarly, biomimetic structures inspired by the Saharan silver ant, which uses triangular, hair-like structures for cooling, have been replicated using silk.^57^ This “green” approach, however, harbors a central contradiction. Biopolymers like cellulose and silk are inherently hydrophilic.^58^ Their structural integrity and optical properties are highly susceptible to water ingress, meaning their performance degrades severely in the very humid, wet environments where cooling is often most needed.^59^ Overcoming this “green material paradox” by developing effective, scalable hydrophobic functionalization is a primary frontier for this class of materials.^60^

The quantitative differences and qualitative arguments underpinning these design strategies are summarized in Tables 1–3, but the comparison should be read as a technology-readiness analysis rather than a ranking by a single cooling number. Table 1 compares optical and thermal performance, Table 2 links fabrication routes to cost and area scalability, and Table 3 identifies the unresolved tradeoffs that prevent any one class from being universally superior. The central conclusion is that laboratory cooling power, manufacturing compatibility, polymer durability, and field tolerance must be evaluated together; otherwise a material can appear excellent in a bibliography while remaining unsuitable for deployment.

**Table 1:** Comparative performance of representative PRC material classes

Material class	Example material/structure	Avg. Solar reflectance (*R*_sol_)	Avg. Thermal emittance (*ε*_atm_)	Reported cooling performance (under sunlight)	Reference(s)
Photonic metamaterial	HfO_2_/SiO_2_ multilayer stack on Ag	0.97	0.96	≈4.9 °C sub-ambient; 40.1 W m^−2^	30
Scalable metamaterial	Randomized SiO_2_ microspheres in PMMA on Ag	0.96	0.93	≈93 W m^−2^ net cooling power	3
Nanocomposite paint	BaSO_4_ nanoparticles in acrylic binder	0.98	0.95	≈4.5 °C sub-ambient; 117 W m^−2^	61
Nanocomposite paint	CaCO_3_ nanoparticles in acrylic binder	0.955	0.94	≈1.7 °C sub-ambient; 37 W m^−2^	24
Porous polymer film	Hierarchical porous PMMA (no metal)	0.95	0.98	≈6.0–8.9 °C sub-ambient; 85 W m^−2^	47
Biopolymer	Delignified “cooling wood”	0.92	0.96	≈8 °C sub-ambient; 60 W m^−2^	42

**Table 2:** Analysis of fabrication techniques, cost, and scalability

Fabrication method	Material class	Pros	Cons	Scalability & cost	Reference(s)
Physical vapor deposition (PVD)/sputtering	Photonic metamaterials	Unmatched nanometer precision. High-purity, high-performance layers	Extremely slow batch process. Requires high vacuum. Capital-intensive equipment	**Very low scalability.** Prohibitively high cost for large-area applications (*e.g.*, buildings)	62
Photolithography/E-beam lithography	Photonic metamaterials (patterned)	Precise, arbitrary sub-wavelength pattern generation (*e.g.*, pillars)	Multi-step, complex process. Uses toxic resists and etchants. Extremely slow	**No scalability.** Suitable only for laboratory prototypes and microchips	63
Roll-to-Roll (R2R) processing	Scalable metamaterials, polymer films	High-throughput, continuous manufacturing. Economical for large-area films	Lower precision than PVD. Limited material compatibility	**High scalability.** The gold standard for producing low-cost, large-area flexible photonic films	3
Phase inversion/solution casting	Porous polymers, nanocomposites	Simple, low-energy process. Self-assembles porous structures. Scalable batch or R2R	Requires large volumes of solvents (environmental/cost issue)	**High scalability.** A standard industrial process for polymer membrane production	64
Spray coating/blade coating	Nanocomposite paints	Extremely fast and low-cost. Compatible with existing paint infrastructure. Can coat complex, non-flat surfaces	Poor thickness/uniformity control. Performance can be inconsistent	**Massively scalable.** The most practical and economical method for deployment	46
Delignification/chemical treatment	Biopolymers (cooling wood)	Uses abundant, low-cost biomass feedstock	Batch chemical process. Requires significant water and chemical (*e.g.*, NaOH, Na_2_SO_3_) inputs	**Moderate scalability.** Potential for industrial scale but less mature than polymer processes	65

**Table 3:** Summary of the central scholarly debate: performance *vs.* scalability

Viewpoint	Core argument	Supporting evidence (key papers)	Main counter-argument/limitation
Performance-first (photonic metamaterials)	Achieving theoretical-limit performance is paramount. Precise spectral control *via* nanophotonics is the only way to maximize cooling power, especially in challenging climates	Multilayer HfO_2_/SiO_2_ stacks demonstrating deep sub-ambient cooling^29^	Fabrication methods (PVD, lithography) are far too expensive and slow for large-scale, real-world deployment
Scalability-first (nanocomposite paints)	Cost and ease of deployment are the only metrics that matter for global impact. A “good enough” paint that can be rapidly deployed is superior to a “perfect” lab-scale chip	Development of BaSO_4_ and CaCO_3_ paints using standard paint processes, demonstrating viable cooling at massive scale and low cost^40^	Performance is inherently compromised. High particle loading can degrade mechanical durability and IR emission
The resolution (scalable metamaterials)	Performance and scalability are not mutually exclusive. Disordered or randomized photonics can achieve near-ideal performance using low-cost, high-throughput manufacturing	Randomized glass-polymer hybrid (SiO_2_ spheres) achieving 93 W m^−2^ cooling power while being fabricated *via* scalable roll-to-roll processing	Still relies on a metal back-reflector (Ag), which is vulnerable to oxidation and increases cost/complexity compared to single-layer paints
The alternative (sustainable biopolymers)	The entire debate over photonics and paints ignores the looming crisis of material sustainability and end-of-life. The solution must be biodegradable, non-toxic, and from an abundant feedstock	Engineered “cooling wood” and cellulose-based films demonstrating high-performance cooling using an inherently sustainable and nanostructured material^66^	Biopolymers are hydrophilic and have poor water resistance, compromising both mechanical and optical performance in humid/wet weather^59^

## 4. Application advances and real-world performance challenges

The diverse material strategies for passive radiative cooling are being pursued to serve a rapidly expanding range of applications, from cooling entire cities to individual people.^67^ The most significant and largest-scale application is in the built environment (Table 4). Applying PRC coatings to building envelopes (roofs and facades) offers a direct pathway to reducing global energy consumption for space cooling.^68^ Simulations and field studies have shown that high-performance PRC materials can drastically reduce the solar heat gain of a building, lowering daytime surface temperatures and reducing the subsequent heat flux into the conditioned space.^69^ This, in turn, lessens the operational load and run-time of active AC systems, with projected energy savings ranging from 20% to 60%, depending on the climate and building stock.^55^ Beyond single-building savings, the widespread adoption of PRC “cool roofs” has profound implications for urban climatology.^70^ By reflecting sunlight and radiating heat out of the troposphere, PRC surfaces can directly combat the UHI effect, lowering ambient air temperatures in cities and breaking the feedback loop of urban overheating.^71^

**Table 4:** Case studies of PRC field-testing in extreme climates

Location	Climate type	Material used	Key challenge identified	Observed performance	Reference(s)
Hong Kong	Hot, humid subtropical	Selective radiative-cooling surface	High atmospheric water vapor and strong downward IR radiation	Cooling remained useful relative to conventional surfaces, but daytime sub-ambient cooling was humidity-limited	82
Tropical daytime limit analysis	Hot, humid tropical	Idealized and practical PRC emitters	High PWV narrows the effective sky window and raises apparent sky temperature	Sub-ambient cooling becomes thermodynamically difficult when PWV raises the apparent sky temperature	83 and 84
Singapore	Hot, humid tropical	Highly reflective polymeric coating	Humidity, solar irradiance, and convective heat gain	Sub-ambient cooling was achieved, but only with high reflectance/emittance and favorable test conditions	85
West Lafayette, IN, USA	Temperate clear-sky baseline	BaSO_4_-acrylic paint	Benchmark condition for high-reflectance scalable paint	Sub-ambient cooling and high net cooling power were demonstrated under favorable outdoor conditions	61
Arizona, USA (arid)	Hot, arid desert	PRC-assisted building/water systems	Dust, soiling, and water availability become dominant engineering concerns	System studies indicate large potential, but maintenance and dust management remain critical	8 and 80

At the other end of the spectrum, PRC principles are being applied to personal thermal management (PTM) through advanced textiles. Human skin is an efficient radiator, emitting thermal radiation peaked at a wavelength of approximately 9.5 µm, which falls squarely within the 8–13 µm atmospheric window.^72^ Conventional textiles like cotton or polyester are opaque to this mid-infrared radiation, trapping the body's heat.^73^ PRC textiles are engineered to be “infrared transparent”. Materials like nanoporous polyethylene (nanoPE), engineered silk, and cellulose acetate are designed with pore structures or molecular compositions that allow this bodily IR radiation to pass directly through the fabric, enabling a new, passive cooling mechanism.^74^ When combined with high solar reflectance to block incoming heat, these textiles can significantly improve thermal comfort outdoors without any energy input.^75^

PRC is also a critical enabling technology for other energy and water systems, particularly in extreme-weather environments. Photovoltaic (PV) panels, for example, suffer from a significant performance drop in hot climates. Their efficiency degrades as their operating temperature rises; for silicon cells, this can be a 0.45% efficiency loss for every 1 °C increase.^76^ This is especially problematic in hot, arid regions where high solar irradiance is co-located with extreme ambient temperatures.^77^ By integrating a PRC cooling layer with the PV module, it is possible to lower the panel's steady-state operating temperature by 5–10 °C,^78^ boosting its electrical efficiency and, critically, slowing thermal degradation pathways to extend its operational lifetime.^79^ In a similar vein, the cold sub-ambient surface of a PRC material can be used for atmospheric water harvesting. By cooling a surface below the dew point, even in arid regions with low relative humidity, PRC systems can passively condense and collect fresh water, providing a vital resource for off-grid or drought-stricken areas.^80,81^

Despite this vast application potential, a significant reality gap exists between laboratory performance and real-world viability, especially in extreme climates.^82–84^ Hot-humid field studies show that high precipitable water vapor increases downward atmospheric radiation and narrows the effective infrared escape window; as a result, daytime sub-ambient cooling can become marginal or disappear even when a coating remains much cooler than a conventional roof surface.^82,83^ Singapore field data further show that sub-ambient cooling is still possible under tropical conditions, but only when reflectance, emittance, convective shielding, and sky conditions are all favorable.^85^ Thus, the practical metric in humid climates should not be limited to the maximum sub-ambient temperature drop. For buildings, the more deployment-relevant question is whether the coating lowers heat flux into the envelope over weeks or seasons without losing reflectance through soiling, water uptake, or polymer aging.^86^

Beyond immediate atmospheric performance, the most significant barrier to long-term, large-area PRC adoption is engineering reliability. A material's initial optical properties are useful only if they can be retained for a 10- to 20- year roof or facade service life. Polymeric materials, which form the basis of most scalable designs, face UV photooxidation, moisture exposure, thermal cycling, abrasion, and contamination.^87–91^ For multilayer materials that rely on Ag or Al back-reflectors, moisture and oxygen ingress can tarnish the metal, reduce solar reflectance, and initiate delamination, motivating encapsulation or all-dielectric architectures.^92–94^ The most universal challenge is soiling: dust, pollen, soot, and organic grime add a solar-absorbing layer and can block infrared emission, especially on rough or porous surfaces.^95–97^

At the polymer-chemistry level, degradation proceeds through specific reactions rather than only through visible symptoms such as yellowing or chalking. The choice of functional groups controls UV stability because chromophoric carbonyl, peroxide, unsaturated, or aromatic defects can absorb near-UV photons and initiate photooxidation. For carbonyl-containing binders, Norrish type I cleavage can split the C–C bond adjacent to the excited carbonyl to form acyl and alkyl radicals, whereas Norrish type II reactions involve gamma-hydrogen abstraction followed by chain scission or unsaturated end-group formation.^87,90,110^ The resulting radicals react with oxygen to form peroxy radicals and hydroperoxides, propagating chain scission, crosslinking, carbonyl growth, embrittlement, and visible yellowing. These reactions increase solar absorption and can therefore erase the optical advantage of a high-reflectance coating even before mechanical failure is obvious.

Surface chemistry governs soiling through surface energy, roughness, charge, and water-solid adhesion: hydrophobic low-energy surfaces reduce capillary pinning of dust, whereas photocatalytic TiO_2_-rich surfaces can degrade organic grime but may accelerate polymer aging if radicals attack the binder.^95,98,99^ Robust self-cleaning therefore requires a balanced surface, not simply maximum roughness.^100^ Hierarchical texture must shed contaminants during rain or dew while remaining mechanically durable, and any fluorosilane or other low-energy treatment must survive UV exposure, abrasion, and humidity.^91,98,101^

A final real-world challenge is that a material optimized for extreme heat becomes a liability in extreme cold. A high-emissivity PRC roof that provides “free” cooling in summer will continue to radiate heat in winter, imposing an “overcooling” penalty and increasing the building's heating load.^102^ The ideal solution is not a static material, but a “smart”, adaptive one. This has driven research into dynamic and switchable PRC.^103^ The most-studied approach involves thermochromic materials, such as vanadium dioxide (VO_2_). VO_2_ undergoes a solid-state phase transition at a tunable temperature (*e.g.*, near 68 °C, but dopable).^104^ Below this transition, it is a dielectric with low IR emissivity (heat-trapping “winter mode”). Above it, it becomes metallic and highly emissive in the mid-IR (heat-radiating “summer mode”).^105^ A more recent, novel approach demonstrated by Mandal *et al.*^106,107^ uses a porous polymer that can be reversibly wetted. In its dry state, the air-filled pores (low refractive index) scatter light, making the material white and reflective (“cooling mode”).^108^ When the pores are infiltrated with a refractive-index-matched liquid (*e.g.*, water or a specific solvent), the scattering is eliminated, and the material becomes fully transparent, allowing sunlight to pass through and be absorbed (“heating mode”). These smart, all-season materials represent the next generation of adaptive building skins, designed to respond dynamically to changing weather conditions.^109^

## 5. Conclusion and future outlook

This review shows that passive radiative cooling has moved beyond proof-of-concept optics into a deployment phase where material chemistry and environmental reliability are as decisive as spectral selectivity. The state of the art is therefore not a single material class but a short list of partially mature technology families: ultrawhite nanoparticle paints, hierarchically porous polymer coatings, disordered glass-polymer metamaterials, humidity-tolerant emitters, and switchable all-season skins. Each family solves one part of the problem while exposing a different weakness: paints are scalable but can suffer from binder aging and particle-loading tradeoffs; photonic stacks are spectrally precise but costly and metal-dependent; porous polymers are optically powerful but vulnerable to contamination and moisture; and dynamic systems reduce seasonal overcooling but add material complexity (Table 5).

**Table 5:** Durability and degradation mechanisms in PRC materials

Degradation mechanism	Primary cause	Materials primarily affected	Performance impact	Mitigation strategy	Reference(s)
UV photodegradation and photooxidation	Near-UV absorption by chromophores; Norrish I/II reactions; radical chain scission	Acrylics, polyolefins, EVA, carbonate/ester binders, and poorly stabilized matrices	Yellowing, chalking, embrittlement, carbonyl growth, increased solar absorption, and *R*_sol_ loss	UV-stable backbones, HALS/UV absorbers, inorganic screens, reduced chromophores, and accelerated-aging tests	87, 90 and 110
Metallic oxidation/corrosion	O_2_, H_2_O, and pollutant ingress through defects or edges	Ag or Al back-reflectors in multilayer and hybrid emitters	Reflectance loss, delamination, and irreversible optical failure	Barrier encapsulation, edge sealing, corrosion-resistant reflectors, or metal-free designs	91–94
Environmental soiling	Dust, soot, pollen, salts, and organic grime on rough or high-energy surfaces	All outdoor-facing materials; porous coatings are especially vulnerable	Higher solar absorption, blocked IR emission, and faster cooling-power loss	Balanced self-cleaning, durable low-energy treatments, and photocatalysis with binder protection	95, 98 and 99
Moisture ingress/water resistance	Rain, dew, humidity, and capillary uptake into porous networks	Biopolymers, hydrogels, cellulose/silk structures, and open-pore coatings	Swelling, pore collapse, reduced scattering contrast, and weakening	Hydrophobic functionalization, cross-linking, pore-wall reinforcement, and humidity-specific testing	59, 91 and 101

The main gaps in the literature are now clear. First, humid-climate design remains underdeveloped: many emitters are optimized for the ideal 8–13 µm atmospheric window rather than for the narrower and warmer humid-sky window that dominates tropical deployment. Second, outdoor durability is still reported inconsistently; short tests of reflectance or temperature drop cannot substitute for accelerated UV/humidity/soiling protocols linked to lifetime prediction. Third, surface chemistry is often treated as a finishing step, even though dust adhesion, self-cleaning, water resistance, and polymer photooxidation directly determine retained cooling power. Fourth, there is still a lack of standardized field metrics that compare sub-ambient temperature, heat-flux reduction, building energy savings, maintenance cost, and seasonal heating penalties on the same basis.

Future progress should therefore prioritize integrated design rules rather than isolated hero records. Materials for humid regions should tune emission to the actual atmospheric window available under high precipitable water vapor. Scalable coatings should report not only initial *R*_sol_ and epsilonatm values but also their retention after UV exposure, abrasion, washing, dust loading, and wet-dry cycling. Polymer binders should be selected by combining infrared vibrational emissivity, solar transparency, photochemical stability, surface energy, and compatibility with high particle volume fractions. Finally, adaptive and switchable radiative coolers must demonstrate that their seasonal benefits outweigh their added manufacturing and control complexity. The field will have the greatest climate impact when passive radiative cooling is evaluated as a durable building-envelope technology rather than as a one-day optical experiment.

## Author contributions

J. X. D. and Y. P. W. contributed equally to this work; J. X. D. and Y. P. W. conducted conceptualization, investigation, formal analysis, data curation, and writing – original draft; S. H. M. provided methodology, supervision, project administration, and funding acquisition; J. X. D. and Y. P. W. performed visualization; S. H. M. revised the manuscript for critical intellectual content; all authors participated in writing – review and editing; all authors have read and agreed to the published version of the manuscript.

## Conflicts of interest

There are no conflicts to declare.

## Data Availability

No primary research results, software or code have been included and no new data were generated or analysed as part of this review.
